# Hand Milling Induced Phase Transition for Marcasite-type Carbodiimide

**DOI:** 10.1021/jacs.5c00962

**Published:** 2025-03-24

**Authors:** Yuzuki Yamamoto, Kazuki Kume, Suzuka Miyazaki, Ayako Shinozaki, Peng Song, Sayed Sahriar Hasan, Kenta Hongo, Ryo Maezono, Hiroki Ubukata, Hiroshi Kageyama, Mikio Higuchi, Yuji Masubuchi

**Affiliations:** † Graduate School of Chemical Sciences and Engineering, Hokkaido University, N13 W8, Kita-ku, Sapporo 060-8628, Japan; ‡ Faculty of Science, Hokkaido University, N10 W8, Kita-ku, Sapporo 060-0810, Japan; § Institute of Multidisciplinary Research for Advanced Materials, Tohoku University, 2-1-1 Katahira, Aoba-ku, Sendai, Miyagi 980-8577, Japan; ∥ School of Information Science, JAIST, Asahidai 1-1, Nomi, Ishikawa 923-1292, Japan; ⊥ Research Center for Advanced Computing Infrastructure, JAIST, Asahidai 1-1, Nomi, Ishikawa 923-1292, Japan; # Department of Energy and Hydrocarbon Chemistry, Graduate School of Engineering, Kyoto University, Kyoto 615-8510, Japan; ¶ Faculty of Engineering, Hokkaido University, N13 W8, Kita-ku, Sapporo 060-8628, Japan

## Abstract

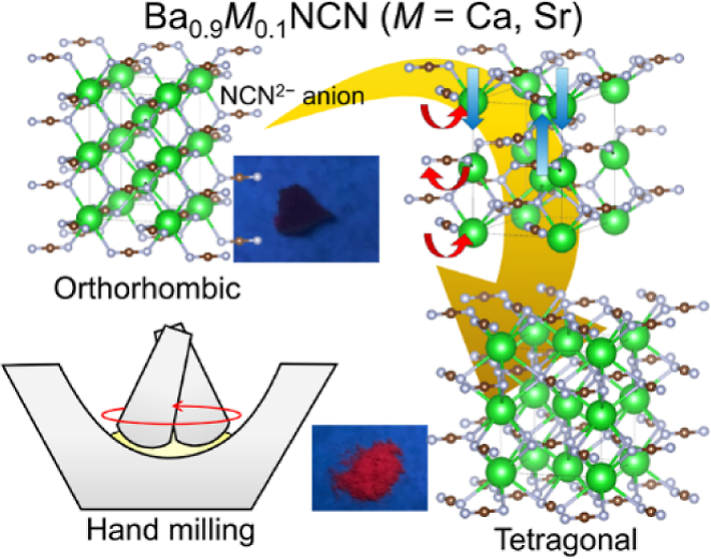

While high-pressure phase transitions have been widely studied in inorganic compounds such as oxides and chalcogenides, relatively little attention has been given to compounds containing molecular anions, such as carbodiimides and cyanamides. This study investigates the phase transition of marcasite-type orthorhombic Ba_0.9_M_0.1_NCN carbodiimides, where a transformation to a CsCl-type tetragonal phase was observed at room temperature under hydrostatic pressure of 0.8 GPa (M = Ca) and 0.3 GPa (M = Sr). This transition, accompanied by an increase in the metal coordination under from 6 to 8, occurs at pressures significantly lower than those required for the high-pressure phase transitions of rock-salt-type metal halides and marcasite-type metal diantimonides. Remarkably, partial phase transitions were also induced by hand milling, a process that applies localized shear forces, distinct from the particle-crushing effects of high-energy ball milling. The transition mechanism, analyzed via variable-cell nudged elastic band (VCNEB) calculations, revealed that while the linear NCN^2–^ anions remain stable, the shear-sliding of Ba^2+^ cations and the rotation of NCN^2–^ anions are critical to the structural transformation. These findings underscore the potential of hand milling to effectively induce phase transitions in compounds containing linear molecular anions, offering new strategies for predicting and controlling such transitions in similar materials.

## 1 Introduction

Pressure plays a pivotal role in driving structural phase transformations in crystalline solids. For instance, body-centered cubic (bcc) iron transforms into a hexagonal close-packed (hcp) structure above 15 GPa, while graphite converts to diamond under 12 GPa.^1,2^ Similarly, ionic compounds such as oxides, chalcogenides, and halides with NaCl-type structures often undergo phase transitions to CsCl-type structures, as observed in FeO at 240 GPa, BaS at 6.5 GPa and NaCl at 27 GPa.^3−5^ These phase transitions typically results in denser polymorphs with higher coordination numbers. In such ionic compounds, the phase stability under pressure can be largely explained by changes in the cation-to-anion size ratio, which critically influences the stability of specific coordination environments.^4,6,7^

Compounds containing molecular anions exhibit unique structural properties under external stimuli.^8−11^ For instance, metal cyanide complexes display highly anisotropic compressibility, including negative linear compressibility.^12−14^ Pressure-induced phase transitions in Zn(CN)_2_ are ascribed to asymmetric displacement of Zn–CN–Zn bridges,^15^ while Zn[Au(CN)_2_] undergoes phase transitions driven by the reorientation of CN^–^ anions within its –NC–Au–CN– framework.^16^ Metal organic frameworks (MOFs) also show remarkable pressure responses,^17^ such as low bulk modulus values (<10 GPa),^18^ pressure induced amorphization,^19^ and phase transitions.^20−22^ For example, (mv)BiBr_5_ (mv = methylviologen) exhibits a phase transition as low as 0.2 GPa due to the reorientation of mv^2+^ molecular cations.^23^ These examples highlight that beyond ionic radius changes, the reorientation and displacement of molecular anions and cations are critical factors driving phase transitions in compounds containing molecular components.

**Figure 1:**
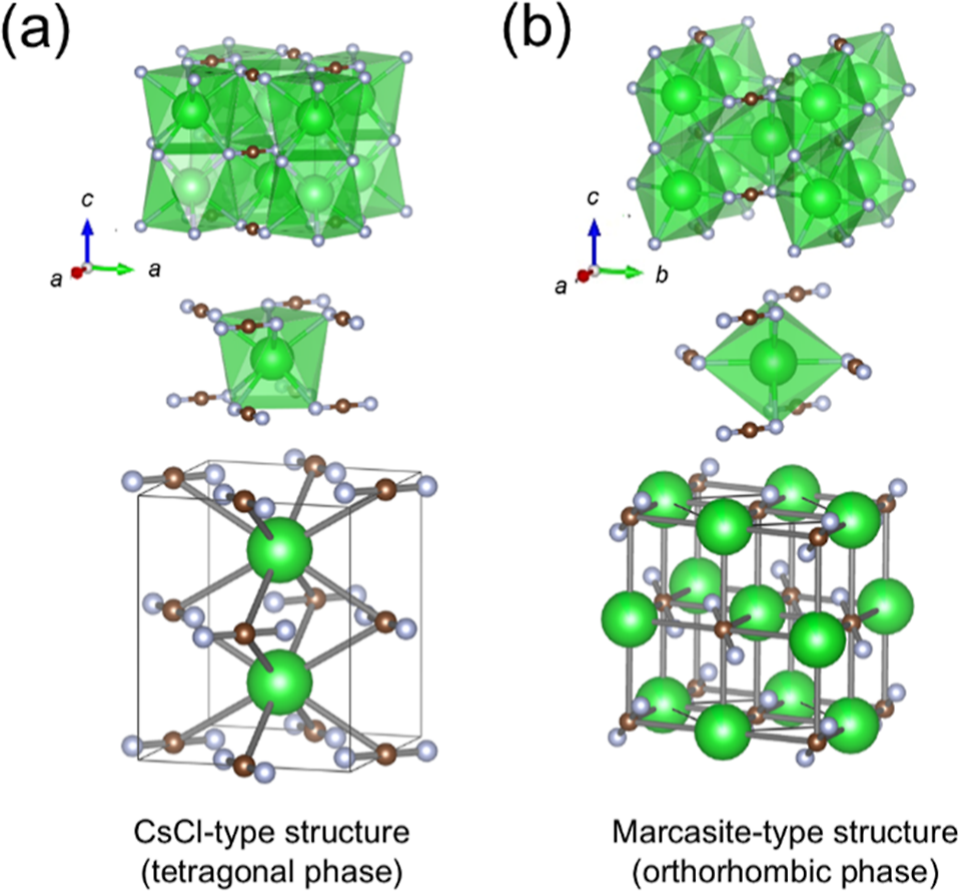
Crystal structures of (a) tetragonal BaNCN having a CsCl-type structure and (b) Ba_0.9_Sr_0.1_NCN having an orthorhombic marcasite-type structure. Green, brown and gray spheres correspond to Ba(Sr), C and N atoms, respectively.

The triatomic NCN^2–^ anion adopts a dumbbell-like linear morphology and carries the same charge as O^2–^, although its covalency is more similar to that of N^3–^.^24^ Compounds containing NCN^2–^ anions have attracted attention for their potential applications in phosphors, catalysts, and battery electrodes.^25−31^ For example, Eu-doped SrNCN and BaNCN exhibit orange and red photoluminescence, respectively,^11,25,26^ while PbNCN, Ag_2_NCN and ZnNCN have been studied for lithium-ion battery electrodes.^30,31^ Despite this interest, structural evolution under pressure has been reported only for PbNCN, HgNCN, BaNCN, and Ag_2_NCN.^32−35^ Müller et al. reported the anisotropic compressibility of PbNCN, due to its layer structure perpendicular to the NCN^2–^ anions.^32^ In contrast, HgNCN decomposes to Hg above 1.9 GPa.^33^ Ag_2_NCN has shown high compressibility up to 23.5 GPa without undergoing a phase transition.^35^ In our previous work, tetragonal BaNCN (Figure 1a) with a CsCl-type structure demonstrated anisotropic compressibility along the *c*-axis up to 7 GPa without a phase transition.^34^

Recently, Ba_0.9_Sr_0.1_NCN, synthesized via ammonolysis of Ba_0.9_Sr_0.1_CO_3_, was reported as a new orthorhombic carbodiimide with a marcasite-type structure (Figure 1b).^36^ Theoretical calculations suggest a pressure-induced phase transition of BaNCN from the orthorhombic marcasite-type to a tetragonal CsCl-type phase (Figure 1a) at 2.8 GPa.^37^ Notably, the marcasite-type carbodiimide has only been experimentally obtained in the Sr-substituted compound, Ba_0.9_Sr_0.1_NCN, under ambient conditions, because of the smaller averaged ionic size of (Ba/Sr)^2+^ than that of Ba^2+^. This study experimentally investigates the pressure-induced phase transition of Ba_0.9_M_0.1_NCN (M = Ca, Sr). In situ synchrotron X-ray diffraction (SXRD) and Raman spectroscopy reveal that the orthorhombic-to-tetragonal phase transition occurs below 1.0 GPa. Computational assessments indicate that this phase transition is of martensitic nature, involving shear sliding of Ba^2+^/M^2+^ cations and rotation of rigid linear NCN^2–^ anions. Unexpectedly, partial phase transition was also induced by hand milling, evidenced by the emergence of red luminescence and changes in Raman spectra and XRD patterns. The relationship between the shear modulus of the carbodiimide and (partial) phase transition during hand milling is discussed.

## 2 Experimental Section

### 2.1 Sample Preparation

The marcasite-type Ba_0.9_M_0.1_NCN (M = Ca, Sr) specimens used in this work were synthesized via the ammonolysis of Ba_0.9_M_0.1_CO_3_ precursors under ammonia, following a previously reported method.^36^ The carbonate precursors were prepared using a complex polymerization process involving BaCO_3_ (99.9%, Fujifilm Wako Pure Chemical Co.), SrCO_3_ (99.9%, Fujifilm Wako Pure Chemical Co.), CaCO_3_ (99.9%, Fujifilm Wako Pure Chemical Co.), citric acid (>98.0%, Fujifilm Wako Pure Chemical Co.) and propylene glycol (>98.0%, Fujifilm Wako Pure Chemical Co.).^38^ For nitridation, the precursors were treated at 900 °C for 15 h under a 100 mL/min NH_3_ flow. Depending on the metal additive, the process included C_3_N_4_ (for M = Ca) or omitted it (for M = Sr). The C_3_N_4_ used in this process was prepared by heating melamine (99.0%, Fujifilm Wako Pure Chemical Co.) at 500 °C for 3 h in air.^39^ A chemical composition of the C_3_N_4_ was determined using an elemental analyzer (Micro Corder JM10, JSL) and the estimated chemical composition was C_3_N_4.5_H_1.5_. The product contained a small amount of hydrogen, although the compound is still referred to as C_3_N_4_ herein. For Ba_0.9_Ca_0.1_NCN, the precursor was mixed with C_3_N_4_ in a 1:4 molar ratio before ammonolysis. To ensure stability, the moisture-sensitive Ba_0.9_M_0.1_NCN products were transferred to an Ar-filled glovebox immediately after synthesis. The Eu-doped Ba_0.9_Sr_0.1_NCN sample was prepared using the same ammonolysis procedure, employing Ba_0.9_Sr_0.1_CO_3_ precursor with 0.5 mol % of Eu added beforehand. Eu acetylacetonate (99.9%, Aldrich) was used as the Eu source. The obtained carbodiimide products were characterized as synthesized.

### 2.2 Characterization

#### 2.2.1 Ambient Pressure Experiments

The crystalline phases of the synthesized products were analyzed at room temperature by powder X-ray diffraction (XRD, Ultima IV, Rigaku) using Cu Kα radiation. To prevent moisture exposure, powder samples were placed in an airtight sample holder in the Ar-filled glovebox prior to analysis. Raman spectra were recorded under ambient conditions using a confocal Raman microscope (XploRA, Horiba) equipped with a 532 nm excitation wavelength. For these measurements, each sample powder was sandwiched between two glass plates and sealed in the Ar-filled glovebox to prevent hydrolysis reaction. The photoluminescence characteristics of the Eu-doped Ba_0.9_Sr_0.1_NCN, both before and after hand milling, were evaluated under ambient conditions using a fluorescence spectrometer (FP-6500, Jasco).

#### 2.2.2 High-Pressure Experiments

In situ high-pressure experiments were performed using a diamond anvil cell (DAC) setup, equipped with a 600 μm diameter anvil culet and a stainless-steel gasket with a sample chamber of 300 μm diameter and 250 μm thickness. Each sample was dispersed in Daphne 7373 oil, which served as both the pressure medium and a stabilizer to prevent decomposition of the carbodiimides during the experiment. Synchrotron XRD (SXRD) patterns were recorded at the BL-18C beamline at the Photon Factory in the KEK facility, Tsukuba, Japan, under pressures ranging from ambient to 5 GPa. The incident X-ray wavelength was 0.62157 Å, with a beam diameter collimated to 100 μm. Pressure calibration was performed using the fluorescence line of a ruby chip included with the sample in the DAC, applying Mao’s equation.^40^ The lattice parameters of each sample were determined by profile fitting using the RIETAN-FP program.^41^

In situ Raman spectra were also obtained using the DAC technique at pressures up to 2 GPa, with pressure intervals of approximately 0.2 GPa. A 532 nm laser with an attenuated power of 5.5 mW was used, with the beam focused on the sample surface to a diameter of around 10 μm. Prior to spectral acquisition, the sample was allowed to equilibrate at the applied pressure for 30 min.

Ex situ high-pressure experiments were conducted using a multianvil press at 1 and 5 GPa without heating. For these experiments, approximately 200 mg of Eu-doped Ba_0.9_Sr_0.1_NCN was sealed in a boron nitride crucible, enclosed within a carbon tube, and packed in a pyrophyllite cube. After pressure was released back to ambient conditions, the crystalline phases were characterized by XRD. The photoluminescence properties of the Eu-doped Ba_0.9_Sr_0.1_NCN after high pressure treatment were also analyzed using a fluorescence spectrometer (FP-6500, Jasco).

### 2.3 Theoretical Calculations

The phase transition from the original orthorhombic structure to a tetragonal structure was investigated through variable-cell nudged-elastic band (VCNEB) calculations at 0 GPa, implemented in the USPEX code.^42^ In these calculations, the chemical compositions of both the orthorhombic marcasite-type and tetragonal CsCl-type structures was fixed to BaNCN, and cell size was set to 1 × 1 × 2, corresponding to a low-pressure marcasite-type structure with *Z* = 4. Initially, a trajectory was generated between the two phases to map the crystal structures onto one another. This trajectory was then refined using the VCNEB method to identify the minimum energy pathway. The calculations produced 45 intermediate structures, along with the initial and final structures, yielding a total of 47 structures in the results. The climbing and descending images were used to locate the highest energy transition state (TS). In addition, radial distribution functions were estimated for the intermediates, initial, and final structural models. Crystal structure images were visualized using the VESTA program.^43^

## 3 Results

### 3.1 In Situ Observation of Phase Transition

Orthorhombic marcasite-type Ba_0.9_M_0.1_NCN (M = Ca, Sr) was synthesized via ammonolysis, as confirmed by the XRD patterns shown in Figure S1 of the Supporting Information. For Ba_0.9_Sr_0.1_NCN, the lattice parameters were determined to be *a* = 5.515(2) Å, *b* = 6.443(1) Å, and *c* = 4.240(1) Å, in good agreement with previous reports.^36^ Similarly, the XRD pattern of Ba_0.9_Ca_0.1_NCN was indexed to an orthorhombic lattice with slightly reduced lattice parameters (*a* = 5.504(4) Å, *b* = 6.413(2) Å, and *c* = 4.224(2) Å), reflecting the smaller ionic radius of Ca^2+^ relative to Sr^2+^.^44^ Raman spectra collected under ambient conditions (Figure S2) further confirmed the orthorhombic structure. A prominent peak at around 1250 cm^–1^, associated with the symmetric vibration of NCN^2–^ anions, was consistent with prior observations for AENCN compounds (AE = Mg, Sr or Ba).^45^ In addition, the Raman band around 210 cm^–1^, characteristic of orthorhombic marcasite-type carbodiimides, was distinct from the split bands observed in the tetragonal CsCl-type BaNCN structure.

**Figure 2:**
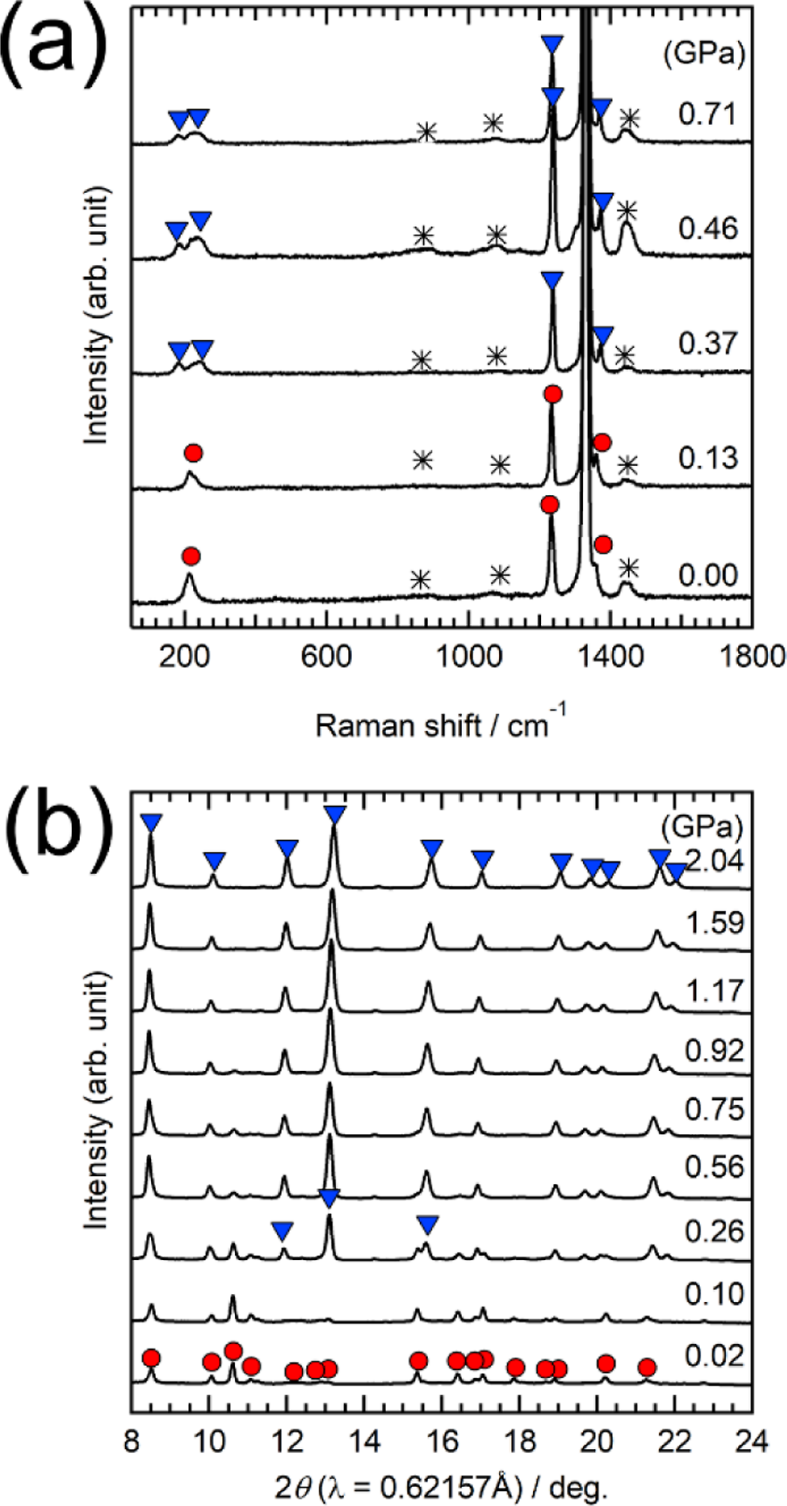
Pressure-dependent (a) Raman spectra and (b) SXRD patterns of Ba_0.9_Sr_0.1_NCN. Red circles indicate the orthorhombic phase, while blue triangles correspond to the tetragonal phase. Asterisks denote Raman shifts associated with the pressure medium and the diamond anvil cell.

The in situ Raman spectra for Ba_0.9_Sr_0.1_NCN under high-pressure compression are shown in Figure 2a. At ambient pressure, a Raman band was observed at around 210 cm^–1^. As pressure increased above 0.37 GPa, this band split into two broader peaks around 190 and 250 cm^–1^. Due to the lower energy resolution of Raman measurements using the DAC technique, only these two Raman peaks near 200 cm^–1^ were detectable at higher pressures. The observed peak splitting is a clear indication of a phase transition from the orthorhombic marcasite-type phase to the tetragonal CsCl-type high-pressure phase. Interestingly, after releasing the pressure, the specimens no longer exhibited the original Raman spectra, suggesting that the high-pressure phase was quenched upon returning to ambient pressure.

The in situ SXRD (Figure 2b) showed the emergence of diffraction peaks corresponding to the tetragonal CsCl-type phase above 0.26 GPa, coexisting with those of the orthorhombic marcasite-type phase. The phase transition to the tetragonal phase was completed at 0.92 GPa. To examine the influence of cation size on the phase transition, in situ Raman and SXRD measurements were conducted for orthorhombic Ba_0.9_Ca_0.1_NCN (Figure S3). The pressures at which the high-pressure phase appeared, as determined by both techniques, are summarized in Table 1. The estimated phase transition pressures, averaged from the two methods, were approximately 0.8 GPa for M = Ca and 0.3 GPa for M = Sr. These results represent the first demonstration of pressure-induced phase transitions in metal carbodiimides with marcasite-type structures.

**Table 1:** Pressures (GPa) at Which the High-Pressure Phase Appeared for Ba_0.9_M_0.1_NCN, Determined by Raman Spectroscopy and SXRD Analyses

	Raman	SXRD	Ave
M = Ca	0.67	0.86	0.8
M = Sr	0.37	0.26	0.3

**Figure 3:**
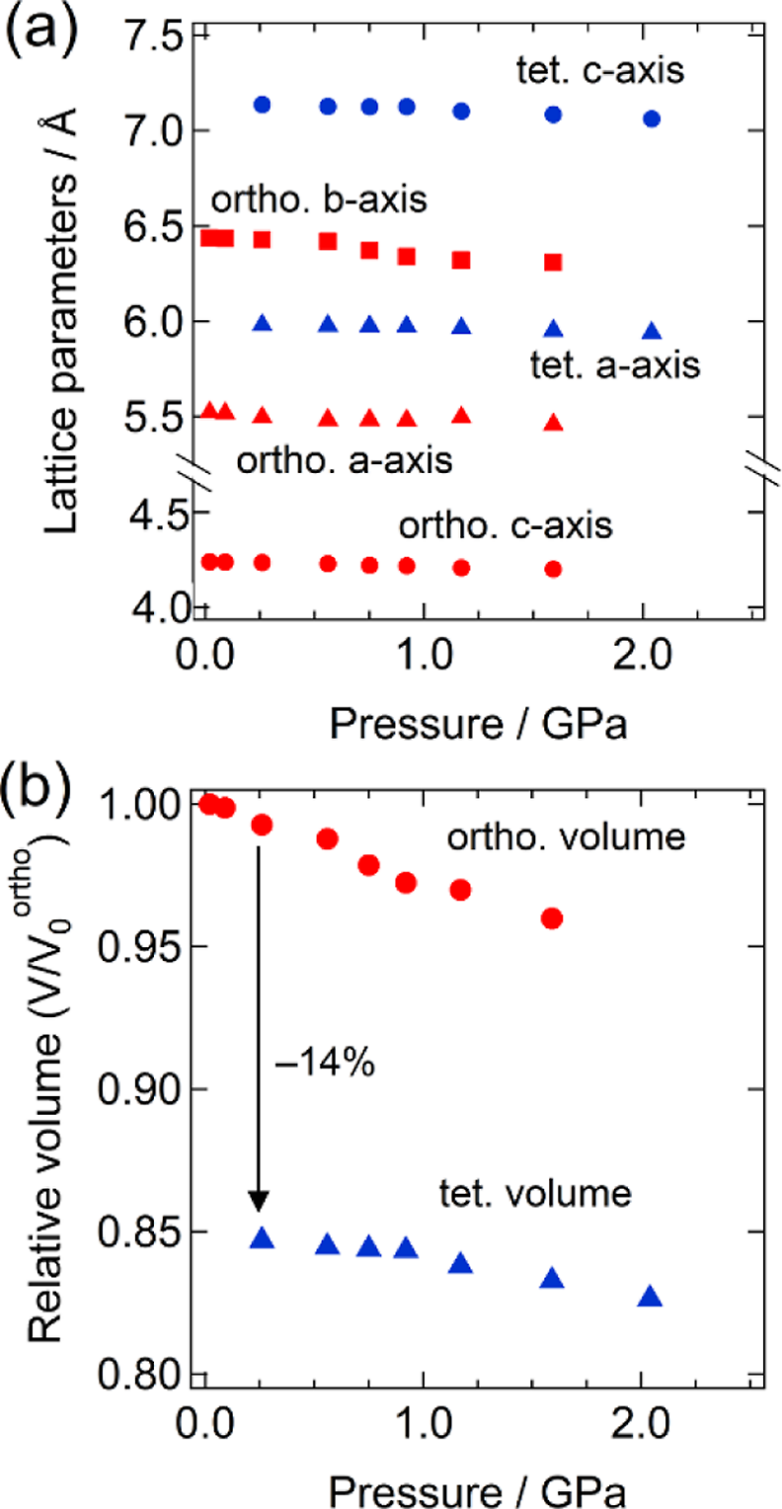
(a) Lattice parameters and (b) relative unit cell volumes of the Ba_0.9_Sr_0.1_NCN as a functions of pressure.

Changes in lattice parameters under high pressure for M = Sr are summarized in Figure 3, revealing anisotropic compressibility along the *b*-axis in orthorhombic Ba_0.9_Sr_0.1_NCN. In the tetragonal phase, the compressibility along the *c*-axis was nearly twice that along the *a*-axis. Pressure-dependent volumes were used to calculate the volume changes associated with the phase transition. For M = Sr (0.3 GPa) and M = Ca (0.8 GPa), the volume reduction was almost identical, around 14% (Figures 3 and S4). The bulk moduli of each compound, both before and after the phase transition, were estimated using the third-order Birch–Murnaghan equation of state.^46^ The values obtained are comparable to those of NaCl- and marcasite-type compounds that undergo a phase transition to the CsCl-type phase above 4 GPa (Table 2).

**Table 2:** Bulk Moduli and Phase Transition Pressures (*P*) for Ba_0.9_M_0.1_NCN (M = Ca, Sr) Compared with Those of Related Compounds Having NaCl-type and Marcasite-type Low Pressure Phase

	*P*/GPa	bulk modulus/GPa	
		low-pressure phase	high-pressure phase
Ba_0.9_M_0.1_NCN, this work			
M = Ca	0.8	39	47
M = Sr	0.3	27	44
BaS^47^	6.5	39	34
BaSe^47^	6.0	43	42
BaTe^47^	4.8	29	28
FeSb_2_^48^	14	75	68
CdSb_2_^49^	9.3^a^	33	
NaHF_2_^b^^50^	4.3	30^c^	34^c^

a The phase transition required high pressure and leaser heating.

b Phase transition from the marcasite-type intermediate (NaHF_2_-II) to CsCl-type high pressure phase (NaHF_2_-III).

c The bulk moduli are estimated from the reported crystal structural data.

### 3.2 Ex Situ High Pressure Experiments

**Figure 4:**
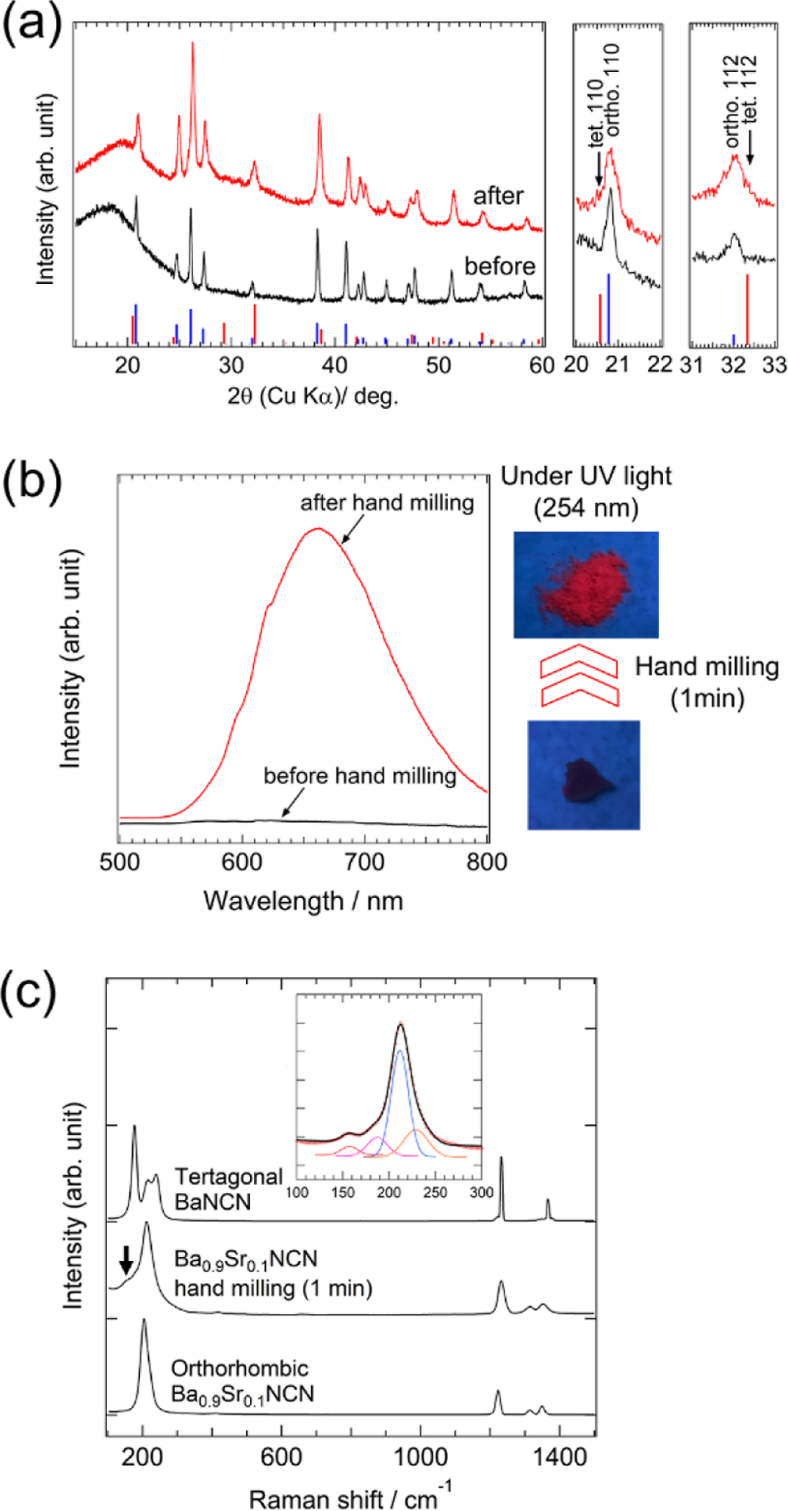
(a) XRD patterns for Ba_0.9_Sr_0.1_NCN:Eu samples before and after 1 min of hand milling. The vertical bars at the bottom indicate the diffraction positions and intensities for the tetragonal (red) and orthorhombic (blue) carbodiimide phases. The magnified images highlight the presence of shoulder peaks assigned to the tetragonal phase. (b) Luminescence spectra of Ba_0.9_Sr_0.1_NCN:Eu before (black) and after (red) hand milling. Emission spectra were acquired with excitation at 267 nm. Photographs show the sample before and after hand milling under 254 nm UV light irradiation. (c) Raman spectra of the as-synthesized orthorhombic Ba_0.9_Sr_0.1_NCN and the hand-milled powders, compared with the tetragonal BaNCN phase. Black arrows indicate additional Raman peaks. The inset shows the peak deconvolution of the Raman spectrum from the hand-milled sample, with peaks attributed to the orthorhombic (blue) and tetragonal (red) phases.

Tetragonal CsCl-type BaNCN doped with Eu^2+^ ions exhibits red luminescence under blue or ultraviolet (UV) light, with the emission wavelength being highly sensitive to temperature and pressure.^11,51^ Eu^2+^-doped orthorhombic Ba_0.9_Sr_0.1_NCN was synthesized via ammonolysis of Eu-doped Ba_0.9_Sr_0.1_CO_3_. However, this material did not exhibit photoluminescence in the visible light region under blue or UV light irradiation (Figure 4b). To explore photoluminescence properties of tetragonal Ba_0.9_Sr_0.1_NCN:Eu, the phase transition of orthorhombic Ba_0.9_Sr_0.1_NCN:Eu was investigated.

Ex situ high-pressure experiments were performed at ambient temperature using a multianvil press, where orthorhombic Ba_0.9_Sr_0.1_NCN:Eu was subjected to compression. Upon applying a pressure of 1 GPa, the XRD pattern collected at ambient pressure showed the coexistence of tetragonal and orthorhombic phases (Figure S5). At 5 GPa, the structure was fully changed to the tetragonal phase, with lattice parameters *a* = 6.002(1) Å and *c* = 7.155(2) Å. These values were slightly smaller than those of tetragonal BaNCN due to the partial substitution of smaller Sr^2+^ (and Eu^2+^) ions for Ba^2+^ ions.^44^ Interestingly, the tetragonal Ba_0.9_Sr_0.1_NCN:Eu obtained at 5 GPa exhibited red photoluminescence at 660 nm upon 480 nm irradiation (Figure S6). These observations confirm that the orthorhombic carbodiimide undergoes a pressure-induced phase transition to the tetragonal phase, in agreement with the in situ results described earlier.

Ex situ high-pressure experiments using a multianvil cell demonstrated that the high-pressure tetragonal structure was retained at ambient pressure following compression to 5 GPa (Figure S7a). Moreover, this high-pressure phase was fully reverted to the orthorhombic marcasite-type structure by thermal annealing at 450 °C in an Ar flow (Figure S7b). These results indicate that the high-pressure phase is kinetically stable at ambient pressure and strongly suggest that the phase transition in these carbodiimides is of first-order nature,^52^ despite the structural correlation between the two phases being addressed below. This conclusion is further supported by the discontinuous change in lattice volume observed during the phase transition, as highlighted in the in situ SXRD results above.

### 3.3 Phase Transition Induced by Hand Milling

The orthorhombic marcasite-type Ba_0.9_Sr_0.1_NCN doped with Eu was synthesized via ammonolysis (Figure 4a). Initially, this material showed no photoluminescence under blue or UV light, as shown in Figure 4b. However, unexpectedly, simple hand milling of the orthorhombic sample using an agate mortar and pestle induced red luminescence upon UV irradiation at 254 nm (Figure 4b). This effect was visually evident, as the initially nonluminescence powder gradually transformed into a red luminescence powder during milling, as demonstrated in Movie S1 of the Supporting Information. The hand-milled powder exhibited red luminescence at 660 nm under UV light (Figure 4b), resembling the photoluminescence properties of Eu-doped tetragonal BaNCN and the high-pressure phase of Ba_0.9_Sr_0.1_NCN:Eu. This strongly suggests that the high-pressure phase may have been at least partially induced during the hand milling process.

XRD analysis of the hand-milled Ba_0.9_Sr_0.1_NCN sample revealed a shoulder peak at 21° and peak broadening at around 32°, indicative of a phase transition to the tetragonal phase (Figure 4a). Although the peak broadening was also attributed to decrease in crystallinity after milling, the phase ratio of the tetragonal phase, calculated from the diffraction pattern, showed that the hand-milled product contained 21 mol % of the tetragonal phase and 79 mol % of the original orthorhombic phase (Figure S8). Raman spectroscopy supported this observation: the original orthorhombic sample exhibited a peak at around 210 cm^–1^ (Figure 4c), whereas the hand-milled powder displayed additional peaks, consistent with the spectrum of the tetragonal BaNCN phase, as shown in the inset of Figure 4c. This set of observations—the emergence of red luminescence, the additional Raman peaks, and the XRD shoulder peaks—clearly demonstrate that a partial phase transition can be successfully triggered by hand-milling. Reproducibility of the partial transition during hand milling has been confirmed by three different researchers. Further hand milling for 30 min decreased crystallinity of the Ba_0.9_Sr_0.1_NCN significantly and finally changed the carbodiimide to XRD amorphous, similar to the amorphization of MOFs by ball milling.^53^

## 4 Discussion

This study represents the first systematic demonstration of pressure-induced phase transitions in metal carbodiimides, focusing on Ba_0.9_M_0.1_NCN (M = Ca and Sr) with marcasite-type structures. It sheds light on their structural adaptability and potential functional applications. The bulk moduli of these carbodiimides are 39 GPa for M = Ca and 27 GPa for M = Sr, comparable to those of BaTe (29 GPa), CdSb_2_ (33 GPa), and NaHF_2_ (30 GPa).^47,49,50^ Despite this similarity, the transitions observed here occurred under hydrostatic pressures below 1.0 GPa (0.8 GPa for M = Ca and 0.3 GPa for M = Sr), significantly lower than those in related compounds (see Table 2). This unusual behavior at such low pressures implies the intrinsic lattice instability of the marcasite-type structure, enabling structural rearrangement with minimal applied pressure. Although low-pressure transitions have been reported in MOFs, such as Cu_2_(DB-bdc)_2_dabco and ZIF-4(Zn),^17^ the behavior observed in these carbodiimides is a unique occurrence in inorganic solid-state materials. Unlike MOFs, which typically undergo transitions due to their flexible frameworks, the phase transitions in these carbodiimides likely involve cooperative distortions within their relatively rigid lattice frameworks. Importantly, these phase transitions occur without heating, indicating that long-range atomic diffusion is not involved.

Most importantly, a partial phase transition was observed with simple hand milling using a mortar and pestle, as confirmed by Raman spectroscopy, XRD and red photoluminescence. The pressure applied during hand milling was roughly estimated to be around 10–20 MPa (details in the Supporting Information, Figure S9), which is an order of magnitude lower than the hydrostatic pressures required to induce a phase transition in Ba_0.9_Sr_0.1_NCN (0.3 GPa). This suggests that additional factors contribute to the phase transition during hand milling. Nonhydrostatic pressure and shear stress are known to stabilize high-pressure phases at lower pressures than those required under hydrostatic compression, as observed in PbO and SnO.^54,55^ The shear modulus, which measures a material’s resistance to deformation under shear stress, provides insight into this phenomenon. For example, the orthorhombic marcasite-type BaNCN, which has not been obtained as a single phase compound, has a calculated shear modulus of 15 GPa (details in Supporting Information, Table S1). The value is much lower than that of SnO (30 GPa, calculated by the Materials Project,^56−58^ mp-2097) and comparable to that of PbO (16.8 GPa, mp-19921), both of which exhibit phase transitions under high-energy ball-milling.^54,55^ This indicates that the marcasite-type BaNCN deforms more easily under shear stress, facilitating a phase transition to the tetragonal phase during hand milling with a mortar and pestle.

**Figure 5:**
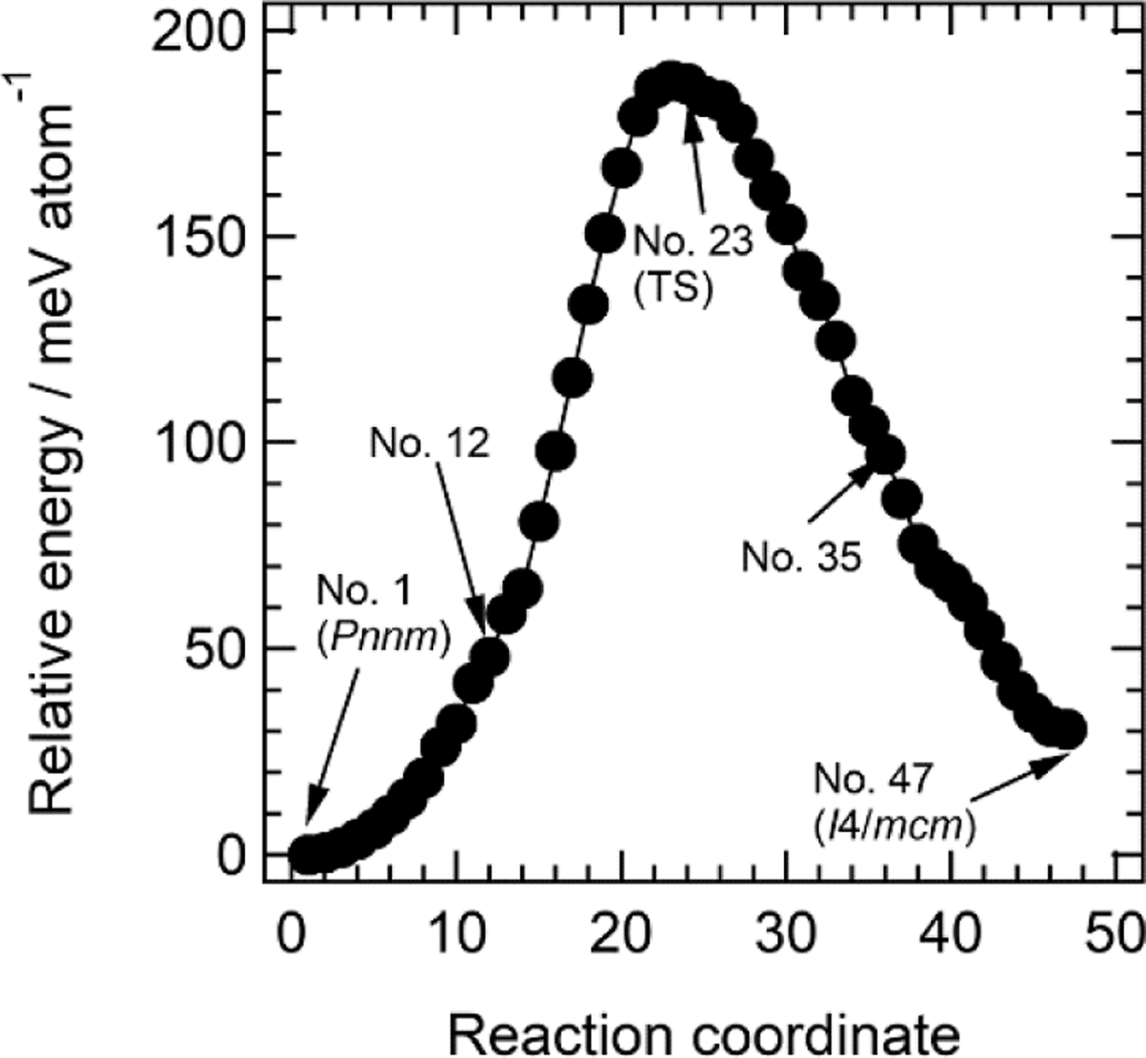
Relative energy of BaNCN during the phase transition from the marcasite-type structure (point no. 1, *Pnnm*) to the tetragonal CsCl-type structure (point no. 47, *I*4/*mcm*). Points 12, 23, and 35 are associated with the intermediate structures shown in Figure 6.

In fact, a comparison between the orthorhombic and tetragonal structures strongly suggests that the phase transition exhibits martensitic characteristics. Atomic displacements during the transition were visualized using VCNEB calculations, with the pressure set at 0 GPa. As shown in Figure 5, these calculations indicate an energy barrier of 188 eV/atom for the transition, which is comparable to the energy barriers for pressure-induced phase transitions in CaCO_3_ and CsPbI_3_,^59,60^ supporting the structural feasibility of the transformation.

**Figure 6:**
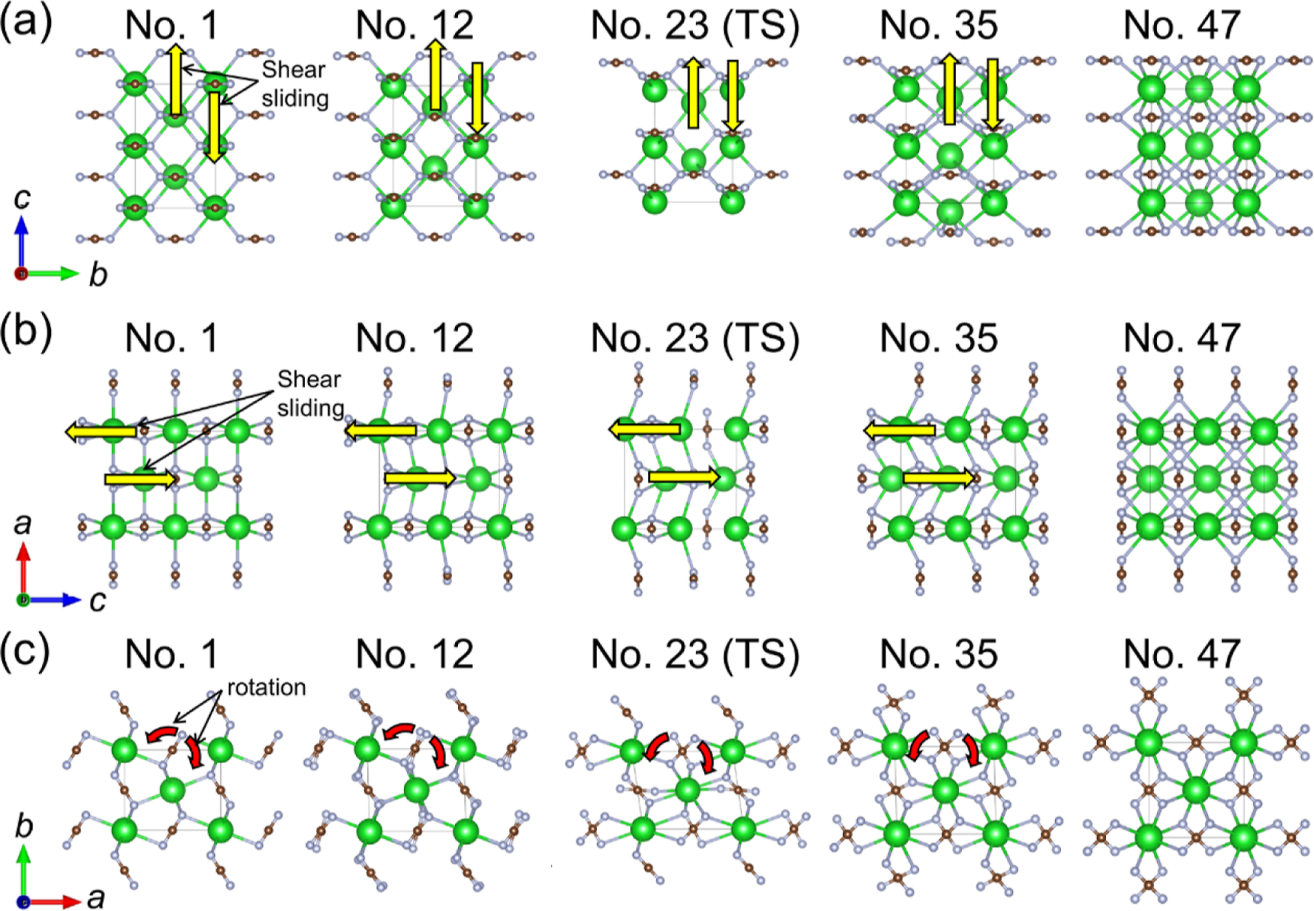
Crystal structures during the phase transition of BaNCN from the orthorhombic phase (point 1 in Figure 5) to the tetragonal phase (point 47 in Figure 5) via a transition state (TS), as generated using the VASP code with the VCNEB method. The crystal structures for the intermediates at points 12 and 35, as well as for the TS at point 23, are shown along the (a) *a*-axis, (b) *b*-axis, and (c) *c*-axis of the orthorhombic lattice. Green, brown, and gray spheres correspond to Ba, C, and N atoms, respectively. Yellow and red arrows indicate the directions of shear sliding of Ba^2+^ cations and the rotation of NCN^2–^ anions, respectively.

As illustrated in Figure 6 and Movie S2 in the Supporting Information, the atomic displacements during the phase transition involve shear sliding of Ba^2+^ along the *c*-axis and rotation of the NCN^2–^ anions. In the final tetragonal structure, the linear NCN^2–^ anions align perpendicularly to their nearest neighbors, while Ba-centered octahedra transform into eight-coordinated square antiprisms. Radial distribution functions (Figure S10) reveal both shorter and longer Ba–N bonds during the transition, stabilizing at around 3.0 Å. The C–N and N–N distances remain almost constant throughout the transition, indicating the robustness of the NCN^2–^ anions’ dumbbell-like geometry (Figure S10c,d).^32^ Recently, tilting and bending of the NCN unit has been observed for the high-pressure form of PbNCN using neutron diffraction, resulting from switching the bond multiplicities in the NCN^2–^ anion.^61^

In contrast to conventional inorganic compounds, where pressure-induced phase transitions are typically driven by changes in the cation-to-anion size ratio, as seen in NaCl-type BaCh and SrCh (Ch = S, Sb, Te),^5^ the transition in these carbodiimides is driven by changes in Ba–N bonding and NCN orientation, via shear sliding of Ba. These findings establish the exceptional nature of carbodiimides as pressure-responsive materials and highlight their unique behavior under shear stress.

Further investigation revealed that AENCN (AE = Ca, Sr) with a layered NaCl-type (*R*3̅*m*) structure (Figure S11) responds differently to hand milling (5 min). Raman spectroscopy detected additional signals in SrNCN, suggesting the onset of a phase transition, while no significant changes were observed in CaNCN (Figure S12). The shear modulus of SrNCN (18.6 GPa) is lower than that of CaNCN (Table S1), supporting the hypothesis that a lower shear modulus facilitates phase transitions under shear stress.

Recent studies have proposed that the shear modulus of starting materials, such as binary oxides and hydrides, can predict the likelihood of shear-force-induced reactions.^62^ For example, BaScO_2_H and SrTiO_3_ perovskites were successfully synthesized via high energy ball milling of the corresponding binary oxides and hydrides. In contrast, the present study found that the martensitic phase transition of carbodiimides can be triggered by hand milling, with the shear modulus of the compounds serving as one of the indicators for the phase transition during milling processes. This finding, together with the computational results obtained in this study, suggests that shear-force-induced phase transitions can be predicted for suitable materials, offering new insights into shear-force-induced reactions in solid-state inorganic materials.

## 5 Conclusions

In this study, phase transitions of marcasite-type Ba_0.9_M_0.1_NCN (M = Ca, Sr) were investigated. When applying hydrostatic pressures, orthorhombic Ba_0.9_M_0.1_NCN transformed to a CsCl-type tetragonal phase at 0.8 GPa (M = Ca) and 0.3 GPa (M = Sr), which are remarkably lower than expected from their bulk modulus. Even more strikingly, partial phase transitions were induced under hand milling, demonstrating the surprising ability of hand milling to trigger phase transitions under much milder conditions. The phase transition process was analyzed using the VCNEB calculations, revealing a martensitic phase transition, where the NCN^2–^ anions remain unchanged, while the rotation of these anions and shear-sliding of Ba^2+^ cations led to an increase in coordination number to eight in the tetragonal structure. These results suggest that shear force during hand milling can be sufficient to induce (partial) phase transitions to the high-pressure phase. This finding opens new possibilities for inducing phase transition in inorganic compounds containing carbodiimide or linear molecular anions, potentially offering a method for predicting such transitions in suitable materials through hand milling.

## Supporting Information Available

XRD patterns of Ba_0.9_M_0.1_NCN (M = Ca, Sr); Raman spectra of Ba_0.9_M_0.1_NCN; in situ Raman spectra and SXRD patterns of Ba_0.9_Ca_0.1_NCN at high pressures; lattice parameters of Ba_0.9_Ca_0.1_NCN at high pressures; XRD patterns of as-synthesized and high-pressure treated Ba_0.9_Sr_0.1_NCN:Eu; PL and PLE spectra of Ba_0.9_Sr_0.1_NCN:Eu after compression at 5 GPa; XRD patterns of pressed and annealed Ba_0.9_Sr_0.1_NCN:Eu samples; radial distribution functions for the selected bonds during the phase transition; estimation of vertical pressure during hand milling; DFT calculation of shear modulus; crystal structure of AENCN (AE = Mg, Ca, Sr); Raman spectroscopy of AENCN before and after hand milling (PDF)
Movie S1. Luminescence of Ba_0.9_Sr_0.1_NCN:Eu power under UV light during hand milling (MP4)
Movie S2. Atomic positions during the phase transition (MP4)
