# Supplementary material for: Hand Milling Induced Phase Transition for Marcasite-type Carbodiimide
Source: J Am Chem Soc. 2025 Mar 24;147(13):11390–8. doi: 10.1021/jacs.5c00962 (PMC11969531; doi:10.1021/jacs.5c00962)
Supplement: Supplementary file 1 — ja5c00962_si_001.pdf [file ja5c00962_si_001.pdf]

Supporting Information

Hand milling induced phase transition for marcasite-type carbodiimide

Authors:

Yuzuki Yamamoto[a], Kazuki Kume[a], Suzuka Miyazaki[a], Ayako Shinozaki[b], Peng Song[c,d], Sayed Sahriar Hasan[d], Kenta Hongo[e], Ryo Maezono[d], Hiroki Ubukata[f], Hiroshi Kageyama[f*], Mikio Higuchi[g], Yuji Masubuchi[g*]

Affiliations:

[a] Graduate School of Chemical Sciences and Engineering, Hokkaido University, N13 W8, Kita-ku, Sapporo, 060-8628, Japan

[b] Faculty of Science, Hokkaido University, N10 W8, Kita-ku, Sapporo 060-0810, Japan

[c] Institute of Multidisciplinary Research for Advanced Materials, Tohoku University, 2-1-1 Katahira, Aoba-ku, Sendai, Miyagi 980-8577, Japan

[d] School of Information Science, JAIST, Asahidai 1-1, Nomi, Ishikawa 923-1292, Japan

[e] Research Center for Advanced Computing Infrastructure, JAIST, Asahidai 1-1, Nomi, Ishikawa 923-1292, Japan

[f] Department of Energy and Hydrocarbon Chemistry, Graduate School of Engineering, Kyoto University, Kyoto 615-8510, Japan

[g] Faculty of Engineering, Hokkaido University, N13 W8, Kita-ku, Sapporo, 060-8628, Japan

*Corresponding Author: Y. Masubuchi, yuji-mas@eng.hokudai.ac.jp; H. Kageyama, kage@scl.kyoto-u.ac.jp

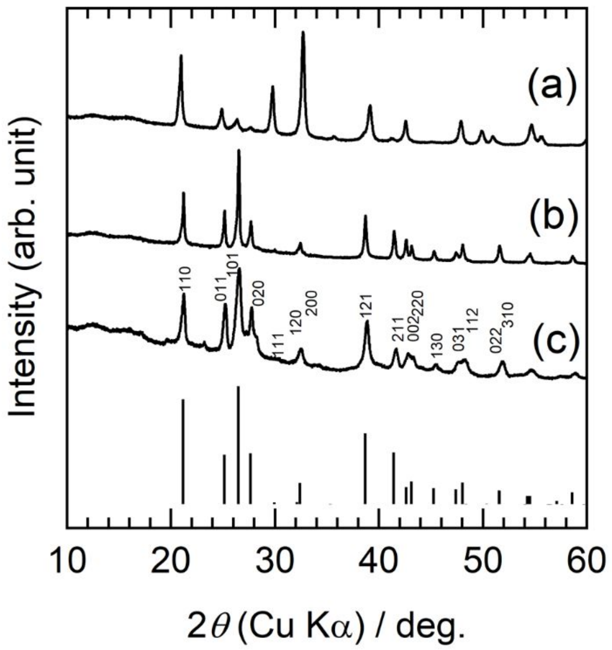

Figure S1. XRD patterns obtained from (a) tetragonal CsCl-type BaNCN, and orthorhombic marcasite-type phases of (b) $Ba_{0.9}Sr_{0.1}NCN$ and (c) $Ba_{0.9}Ca_{0.1}NCN$. The vertical bars at the bottom indicate the peak positions and intensities expected for marcasite-type $Ba_{0.9}Sr_{0.1}NCN$ [S1].

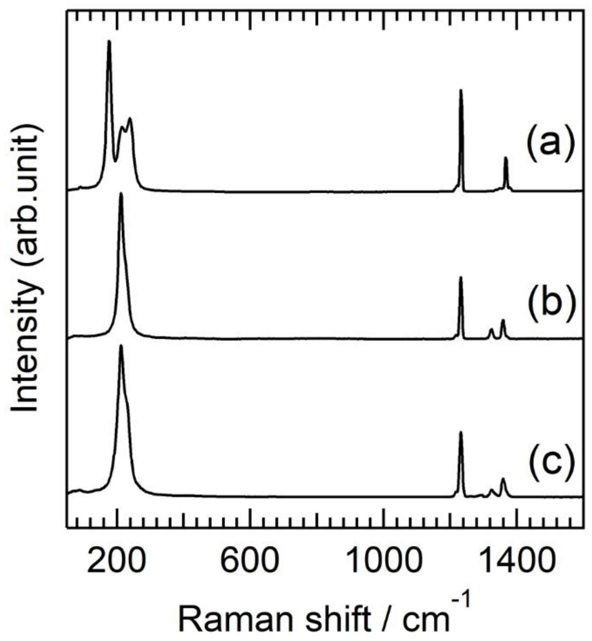

Figure S2. Raman spectra acquired at ambient pressure from (a) tetragonal CsCl-type BaNCN, and as-synthesized orthorhombic marcasite-type phases of (b) $Ba_{0.9}Sr_{0.1}NCN$ and (c) $Ba_{0.9}Ca_{0.1}NCN$.

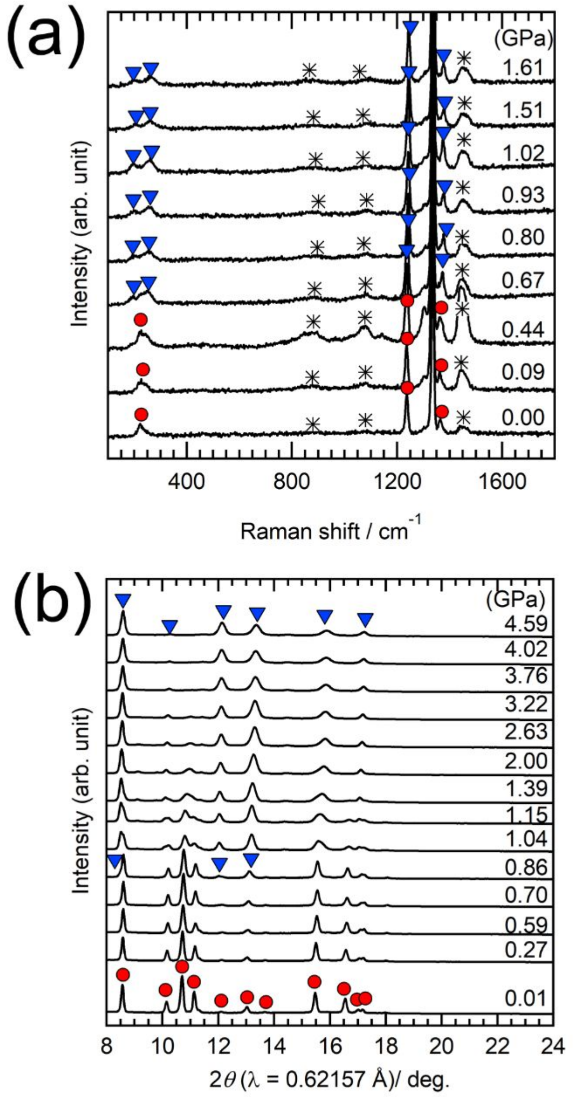

Figure S3. Pressure-dependences of (a) Raman spectra and (b) SXRD patterns for $Ba_{0.9}Ca_{0.1}NCN$ collected using the DAC technique. Red circles indicate the Raman shift and diffraction lines expected for the orthorhombic phase while blue triangles indicate the results expected for the tetragonal phase.

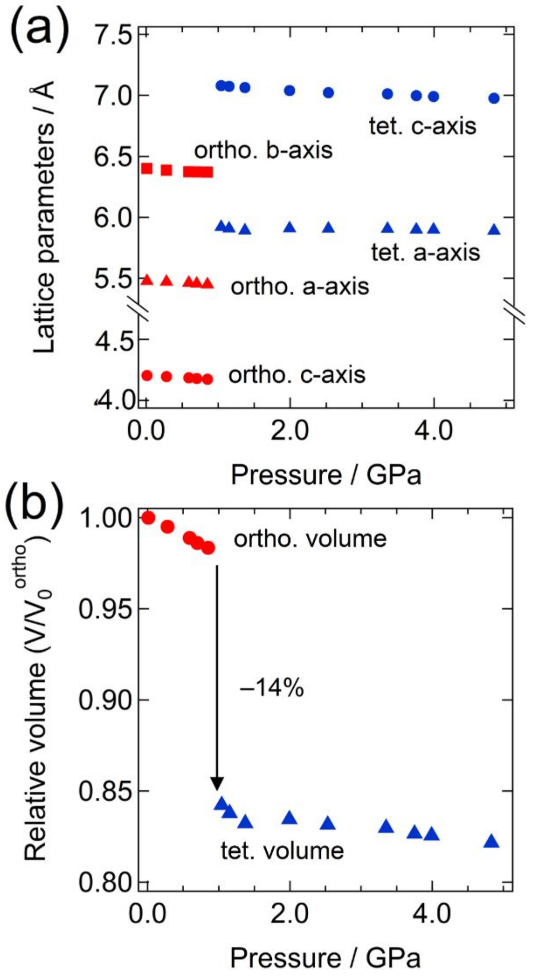

Figure S4. (a) Lattice parameters and (b) normalized unit cell volumes of $Ba_{0.9}Ca_{0.1}NCN$ as a function of pressure.

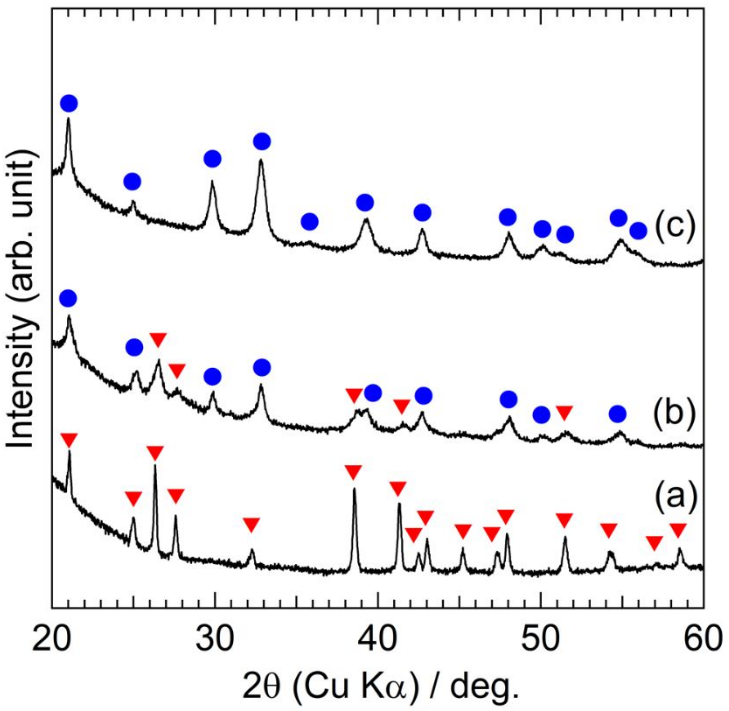

Figure S5. XRD patterns at ambient pressure for (a) the as-synthesized orthorhombic $Ba_{0.9}Sr_{0.1}NCN$:Eu, and specimens after pressing at (b) 1 GPa and (c) 5 GPa using a multi-anvil press. The red triangles and blue circles indicate diffraction peaks attributed to orthorhombic and tetragonal phases, respectively.

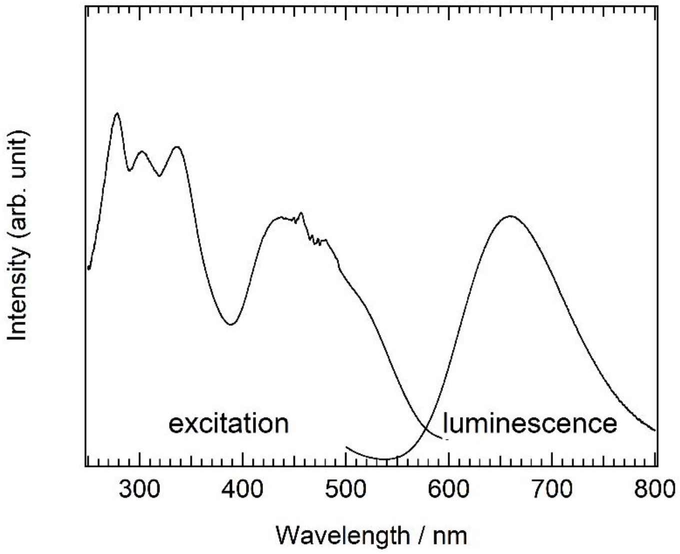

Figure S6. Luminescence and excitation spectra for $Ba_{0.9}Sr_{0.1}NCN$:Eu at ambient pressure after compression at 5 GPa. The emission spectrum was acquired with an excitation of 460 nm and the excitation spectrum was obtained based on luminescence at 660 nm.

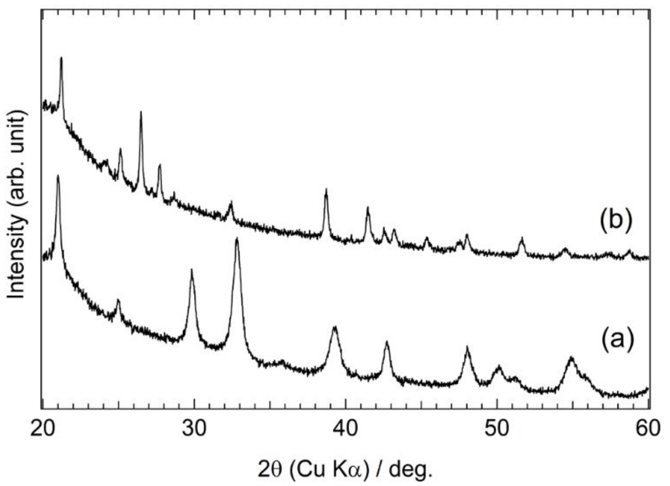

Figure S7. XRD patterns for tetragonal $Ba_{0.9}Sr_{0.1}NCN$:Eu at ambient pressure (a) after pressing at 5 GPa in a multi-anvil press and (b) after annealing at 450 °C for 5 h under an Ar flow.

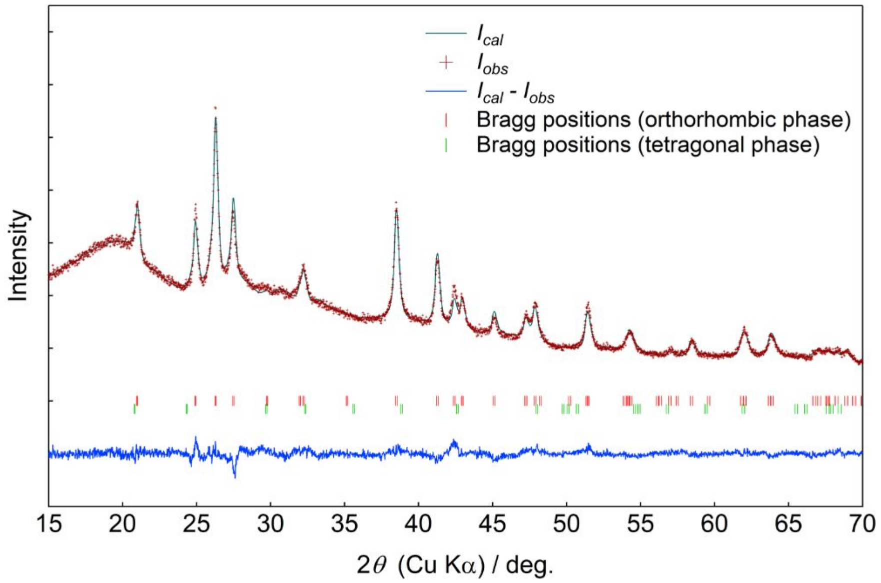

Figure S8. The phase ratio between tetragonal and orthorhombic $Ba_{0.9}Sr_{0.1}NCN:Eu$ phases after hand milling (1 min) by using the XRD pattern. The vertical bars indicate the diffraction positions of tetragonal (green) and orthorhombic (red) phases. The hand-milled sample contained 21 mol% of tetragonal and 79 mol% of orthorhombic phases (R_{wp} = 4.04%, R_p = 2.64%, GOF = 2.45).

Estimation of applied vertical pressure on powder sample during hand milling

Applied force during the hand milling was measured through agate mortar and pestle as shown in Fig. S9. Weights of both the mortar and pestle were subtracted before the measurement. The vertical force was roughly measured to be 1.0-2.0 kg during hand milling with a mortar and pestle. By assuming the contact area between the pestle and surface of the mortar to be 1 mm^2, the applied vertical pressures are estimated to be 10-20 MPa.

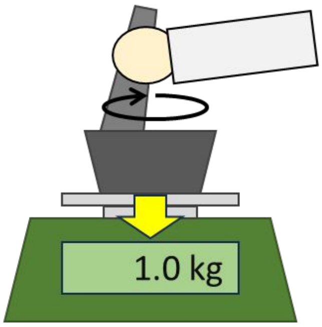

Figure S9. Measurement of the applied force during hand milling.

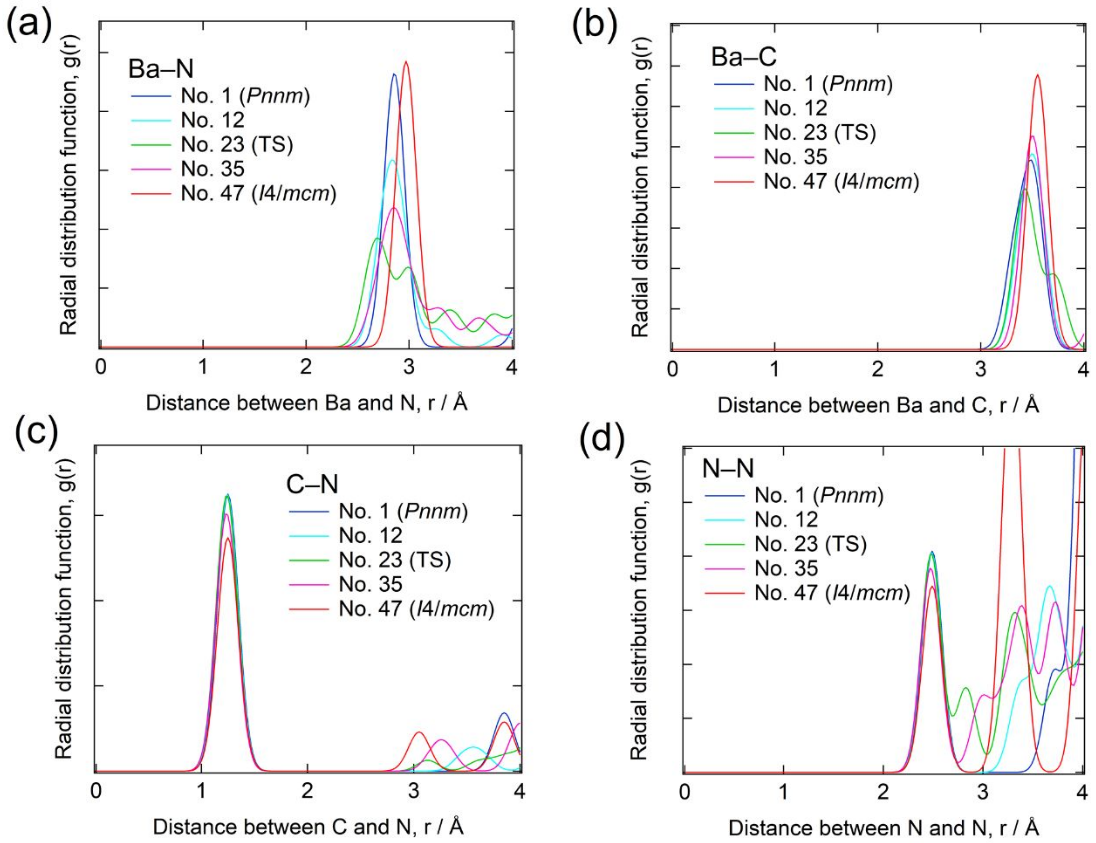

Figure S10. (a) Ba−N, (b) Ba−C, (c) C−N and (d) N−N bond lengths during the phase transition.

DFT evaluation of the shear modulus of the orthorhombic BaNCN

The mechanical properties of orthorhombic marcasite-type BaNCN were calculated as follows: First, the second-order elastic stiffness tensor was obtained using the energy-strain method with the VASP software [S2-S5] and VASPKIT [S6], where the total energies of deformed/strained structures were evaluated at the DFT level. Our DFT calculations employed the GGA-PBE functional [S7] and PAW pseudopotentials, treating the 5s, 5p, and 6s states as valence for Ba, and the 2s and 2p states for C and N. A cutoff energy of 600 eV and an 8 × 8 × 8 Monkhorst–Pack k-point mesh [S8] were sufficient to achieve an accuracy of 1 meV/atom for the total energy. Finally, the ELATE software [S9] was used to evaluate the shear modulus from the computed elastic stiffness tensor. In this calculation, the chemical composition of marcasite-type compound was fixed to BaNCN, instead of $Ba_{0.9}Sr_{0.1}NCN$, because of the limited calculation cost.

Table S1. Averaged shear moduli (GPa) of the carbodiimide compounds in bulk polycrystal

Compounds	S.G.	Shear Modulus / GPa	Ref.
BaNCN	*Pnnm*	15.2	this work
SrNCN	$R\bar{3}m$	18.6	mp-12317
CaNCN	$R\bar{3}m$	25.4	mp-4124

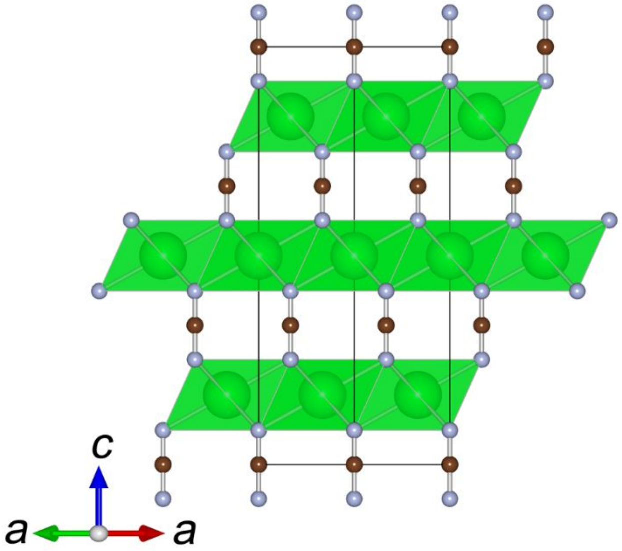

Figure S11. Crystal structure of rhombohedral ($R\overline{3}m$) *AE*NCN (*AE* = Ca, Sr). Green, brown and grey spheres correspond to *AE*, C and N atoms, respectively.

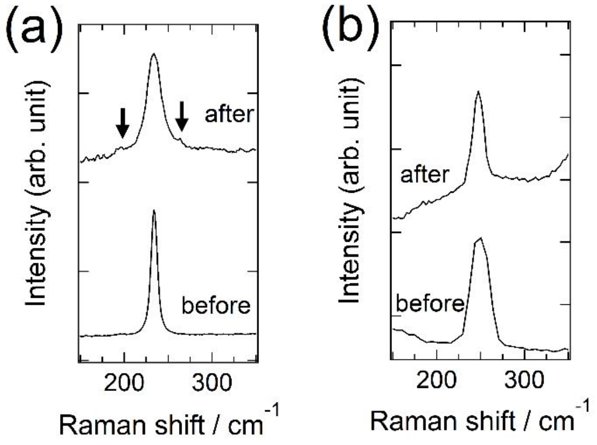

Figure S12. Raman spectra at ambient pressure for *AE*NCN with a rhombohedral layer structure before and after hand milling for 5 min, (a) *AE* = Sr and (b) *AE* = Ca. The arrows indicate the additional Raman signals observed after the hand milling. The phase transition of SrNCN with rhombohedral structure was not clearly observed in its XRD pattern after hand milling. We have preliminary investigated high-pressure phase transition of SrNCN using DAC technique and SrNCN undergo a phase transition to tetragonal CsCl-type structure above 4.0 GPa, which is an order of magnitude higher than that required for marcasite-type $Ba_{0.9}Sr_{0.1}NCN$. Stability of the low-pressure phase in SrNCN suppress the phase transition during hand milling.

SI-movie-1

The original non-luminescence $Ba_{0.9}Sr_{0.1}NCN:Eu$ sample gradually generates red luminescence during hand milling in response to UV light (254 nm).

SI-Movie-2

The mechanism of the orthorhombic to tetragonal phase transition of BaNCN, in which dumbbell-like NCN^{2-} anions rotate to reduce the cell volume and promote the sliding of Ba^{2+} cation interlayers along the *c*-axis.

References

S1. Masubuchi, Y.; Miyazaki, S.; Fujii, F.; Yashima, M.; Miura, A.; Higuchi, M. Ternary carbodiimide compound $Ba_{0.9}Sr_{0.1}NCN$ with distorted rutile-type structure. *J. Solid State Chem.* **2021**, 296, 122000/1-6.

S2. Kress, G.; Hafner, J. *Ab initio* molecular dynamics for liquid metals. *Phys. Rev. B* **1993**, 47, 558-561.

S3. Kress, G.; Hafner, J. *Ab initio* molecular-dynamics simulation of the liquid-metal-amorphous-semiconductor transition in germanium. *Phys. Rev. B* **1994**, 49, 14251-14269.

S4. Kresse, G.; Furthmuller, J. Efficiency of *ab-initio* total energy calculations for metals and semiconductors using a plane-wave basis set. *Comput. Mater. Sci.* **1996**, 6, 15-50.

S5. Kresse. G.; Furthmuller, J. Efficient iterative schemes for *ab initio* total-energy calculations using a plane-wave basis set. *Phys. Rev. B* **1996**, 54, 11169-11186.

S6. Wang, V.; Xu, N.; Liu, J.-C.; Tang, G.; Geng, W.-T. VASPKIT: A user-friendly interface facilitating high-throughput computing and analysis using VASP code. *Comput.*

Phys. Commun. **2021**, 267, 108033/1-19.

S7. Perdew, J. P.; Burke, K.; Ernzerhof, M. Generalized Gradient Approximation Made Simple. *Phys. Rev. Lett.* **1996**, 77, 3865-3868.

S8. Monkhorst, H. J.; Pack, J. D. Special points for Brillouin-zone integrations. *Phys. Rev. B* **1976**, 13, 5188-5192.

S9. Gaillac, R.; Pullumbi, P.; Coudert, F.-X. ELATE: an open-source online application for analysis and visualization of elastic tensors. *J. Phys. Condens. Matter* **2016**, 28, 275201/1-5.
